# 731. Factors Contributing to Long-Term Risk of Developing Multidrug-Resistant *Pseudomonas* Recurrence

**DOI:** 10.1093/ofid/ofad500.792

**Published:** 2023-11-27

**Authors:** Scott Volker, Andrew J Vogler, Kathleen A Fairman, Vanthida Huang

**Affiliations:** Midwestern University, Glendale, Arizona; Midwestern University College of Pharmacy-Glendale Campus, Glendale, Arizona; Midwestern University College of Pharmacy-Glendale Campus, Glendale, Arizona; Midwestern University, College of Pharmacy-Glendale Campus, Glendale, Arizona

## Abstract

**Background:**

Multidrug-resistant *Pseudomonas aeruginosa* (MDR-PA) is a significant healthcare concern in hospitalized patients with a mortality rate upward of 54%. Delayed initiation of appropriate antibiotics with MDR infections may lead to prolonged hospital stays and increased mortality. Previous studies investigated risk factors that contribute to MDR-PA and mortality; however, none evaluated factors leading to recurrence. Therefore, we sought to assess the risk factors contributing to the long-term recurrence of MDR-PA.

**Methods:**

This was a retrospective case-control study of hospitalized patients at HonorHealth Network from January 2015-January 2023. Patients were included if they have ≥2 positive *P. aeruginosa* cultures in 1-year with MDR as first occurrence. Cases were patients with MDR-PA at second occurrence and controls were patients with no MDR-PA at the second occurrence. MDR-PA was defined as resistance to at least 1 antibiotic in ≥3 different antimicrobial classes. Risk factors associated with a secondary MDR-PA occurrence were evaluated using logistic regression backwards-stepwise analysis of clinical signs and symptoms, demographic, admission location, and number of days since the first MDR-PA occurrence.

**Results:**

Of 8,140 hospitalized patients, 209 (n=132 case, n=77 control) met study entry criteria; 52% were female and 84.8% were Caucasian. Respiratory infections (OR 3.13, 95% CI 1.22-8.04), urinary tract infections (UTIs) (OR 4.51, 95% CI 1.82-11.16), intravenous antibiotic use within the past 30 days (OR 3.03, 95% CI 1.05-8.72), and a time period of 30-59 days since the initial culture positive for MDR-PA (OR 4.34, 95% CI 1.37-13.72) were associated with an increased risk of a secondary occurrence of MDR-PA within 1 year.

**Conclusion:**

Our study revealed that there is an increased risk of subsequent recurrence between 30-59 days following the initial MDR-PA positive culture. However, further investigations are warranted in the future to be generalizable.

**Disclosures:**

**All Authors**: No reported disclosures

